# Cellulose supramolecular ionogel with robust mechanical strength and thermal stability for flexible electronics

**DOI:** 10.1002/smo2.70104

**Published:** 2026-09-16

**Authors:** Wenjuan Wang, Minxin Wang, Changhong Lin, Geyuan Jiang, Jianfei Zhou, Dawei Zhao

**Affiliations:** ^1^ Key Laboratory on Resources Chemicals and Materials of Ministry of Education Shenyang University of Chemical Technology Shenyang China; ^2^ Key Laboratory of Leather Chemistry and Engineering (Sichuan University) Ministry of Education Chengdu China; ^3^ State Key Laboratory of Woody Oil Resources Utilization Northeast Forestry University Harbin China

**Keywords:** cellulose, double‐network design, flexible electronics, ionic conductivity, ionogels

## Abstract

Ionogels hold great promise in the fields of flexible electronics and energy devices. However, a persistent challenge lies in the inherent trade‐offs among mechanical strength, ionic conductivity, and thermal stability. Here, we report a double‐network cellulose‐polyvinyl alcohol (Cel/PVA) ionogel via a facile [Emim]BF_4_ displacement regeneration process. The ionogel with a densified supramolecular structure showed a tensile strength of 7.20 MPa, an ionic conductivity of 18.71 mS⋅cm^−1^, a wide electrochemical window of 3.50 V, and outstanding thermal stability up to 135°C. Moreover, flexible sensors fabricated from this ionogel can detect various stimuli, including pressure, temperature, touch, and human pulse signals, producing detectable electrical signal outputs. In addition, the ionogel demonstrates reversible charge‐discharge behavior when tested as an electrolyte in a supercapacitor configuration. This work provides an effective structural design strategy for synergistically optimizing the comprehensive performance of cellulose ionogels, paving the way for their use in next‐generation flexible electronic devices.

## Graphical abstract

In this study, a double‐network cellulose‐polyvinyl alcohol (Cel/PVA) ionogel with a dense supramolecular structure is fabricated via H‐bonding network reconstruction. The Cel/PVA ionogel exhibits outstanding mechanical strength, high ionic conductivity, a wide electrochemical window, and excellent thermal stability, demonstrating its tremendous application in flexible sensing and electronics.

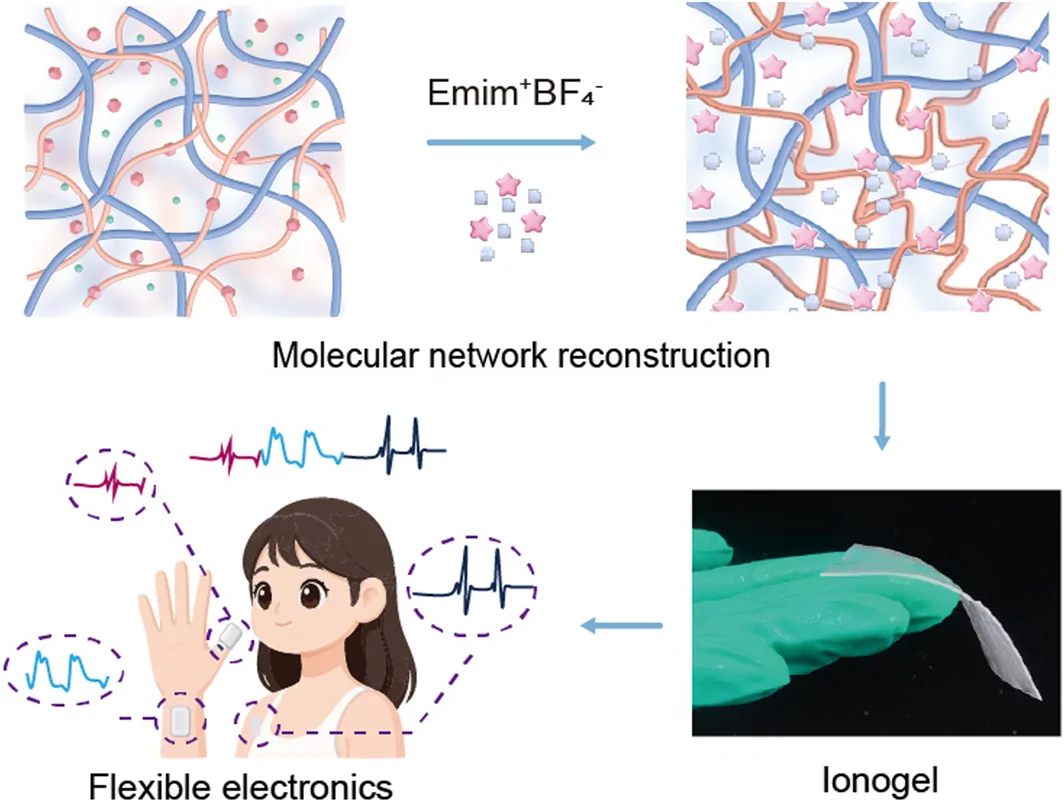

## 1 INTRODUCTION

The rapid iteration of flexible electronics, wearable energy storage devices and intelligent sensing technologies has imposed increasingly stringent requirements on the sustainability, mechanical compatibility and electrochemical performance of core sensing materials.[^1^, ^2^, ^3^, ^4^] As a kind of flexible functional material that integrates the three‐dimensional network structure of gels with outstanding electrochemical properties, ionogels have emerged as highly promising candidate materials for flexible electronic products such as electronic skins,[^5^, ^6^, ^7^, ^8^] intelligent sensors[^9^, ^10^] and energy storage devices,^[11]^ by virtue of their designable microstructures, excellent interfacial compatibility and controllable ion conduction characteristics.

Among numerous gel matrices, cellulose has emerged as an ideal substrate for fabricating high‐performance and sustainable ionogels due to its renewability, biocompatibility, and favorable processability.[^12^, ^13^, ^14^, ^15^, ^16^, ^17^] Nevertheless, cellulose‐based ionogels currently face a persistent performance trade‐off. Although extensive hydrogen‐bond (H‐bond) crosslinking can enhance the mechanical strength and thermal stability of the gels, it tends to trigger local agglomeration and densification of cellulose chains, which severely obstructs internal ion transport channels and consequently sacrifices ionic conductivity.[^18^, ^19^, ^20^, ^21^, ^22^] Furthermore, the strong electrostatic attraction between cellulose chains and free ions further restricts the mobility of charge carriers.[^17^, ^23^, ^24^, ^25^, ^26^] This combined effect results in an inability to balance the mechanical properties, thermal stability, and electrochemical performance of ionogels, thereby severely hindering their application in high‐end flexible devices.

To address the aforementioned limitations, we develop a supramolecular network reconstruction strategy. This method constructs a cellulose/PVA dual‐network structure by introducing PVA molecules. Through a simple and scalable solvent exchange process, a high‐strength, high‐ionic‐conductivity, and high‐thermal‐stability dual‐network cellulose ionogel (Cel/PVA‐EB gel) was successfully fabricated. In this composite system, cellulose acts as a rigid skeleton to guarantee the mechanical strength and stability of the material, while flexible PVA segments serve as reversible energy‐dissipating units. Efficient energy dissipation is achieved through the dynamic dissociation and reformation of the H‐bond between the two components, which greatly improves the mechanical properties and thermal stability of the ionogel.[^27^, ^28^, ^29^, ^30^, ^31^] Subsequently, solvent exchange induces the differential reconstruction of the internal H‐bonding network and electrostatic interactions within the gel. While reinforcing the H‐bonding network, it weakens the electrostatic interactions inside the cellulose skeleton, optimizes ion transport channels, and further enhances ionic conductivity.

The Cel/PVA‐EB gel achieved simultaneous improvements in mechanical, thermal stability, and electrochemical properties. It possesses a tensile strength of up to 7.20 MPa, an ionic conductivity as high as 18.71 mS⋅cm^−1^, an electrochemical window reaching 3.50 V, and thermal stability up to 135°C. In addition, this ionogel shows promising electrochemical behavior in energy storage and intelligent sensing devices, capable of responding to multiple external stimuli with distinguishable electrical signal outputs. In this study, molecular network reconstruction is realized via a simple and efficient solvent replacement strategy, which effectively overcomes the inherent performance trade‐off dilemma of cellulose ionogel. This work opens up a brand‐new avenue for the design and application of high‐performance cellulose ionogel, and simultaneously verifies the broad application prospects of such materials in flexible electronic devices.

## 2 RESULTS AND DISCUSSIONS

### 2.1 Design of cellulose ionogel

**FIGURE 1:**
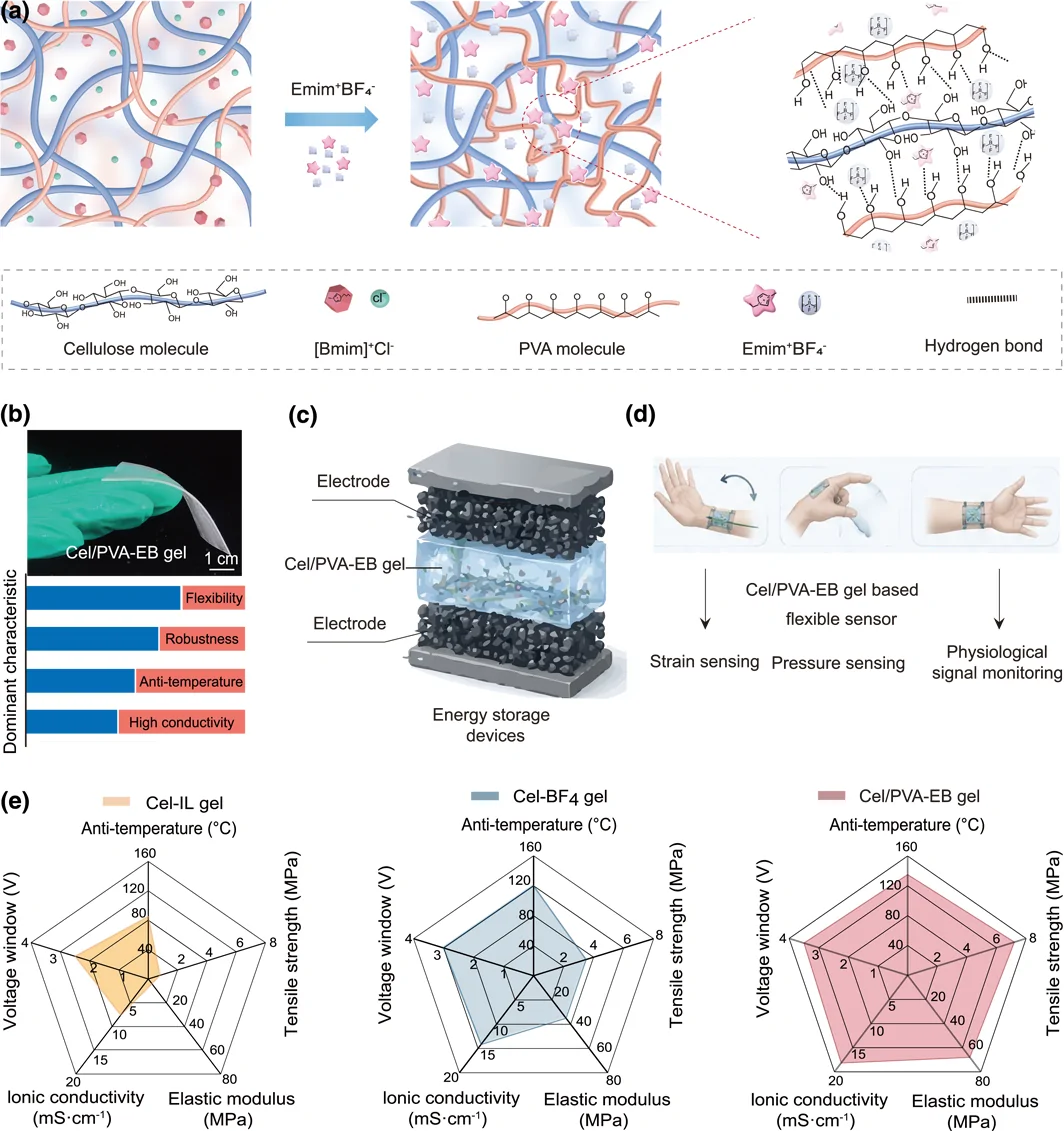
Design and properties of Cel/PVA‐EB gel. (a) Molecular network reconstruction for the development of Cel/PVA‐EB gel. (b) Optical image of the resulting Cel/PVA‐EB gel, highlighting its flexibility, mechanical robustness, thermal stability, and high ionic conductivity. (c) Schematic diagram of the integrated flexible energy storage device using Cel/PVA‐EB gel as the electrolyte. (d) Schematic diagram of the flexible sensing device based on Cel/PVA‐EB gel. (e) Radar plot comparing Cel/PVA‐EB gel with previously reported cellulose ionogel.

First, the ionic liquid 1‐butyl‐3‐methylimidazolium chloride ([Bmim]Cl) was used to disrupt the intermolecular H‐bonding network of cellulose, thereby exfoliating the cellulose molecular chains to form a structural framework. Polyvinyl alcohol (PVA) was then introduced as a flexible component to fabricate a cellulose/PVA ionogel (Cel/PVA‐IL gel). Subsequently, 1‐ethyl‐3‐methylimidazolium tetrafluoroborate ([Emim]BF_4_) was incorporated into the Cel/PVA‐IL gel through solution permeation and molecular thermal diffusion. This process promoted the reconstruction of H‐bonding network and interionic electrostatic interactions, ultimately yielding a dual‐network ionogel (Cel/PVA‐EB gel). Here, “E” and “B” refer to the ethylimidazolium cation ([Emim]^+^) and tetrafluoroborate anion (BF_4_^−^), respectively, derived from [Emim]BF_4_ (Figure 1a).

Significant differences exist between [Bmim]Cl and [Emim]BF_4_ in their H‐bonding competition capacity and electrostatic interactions with cellulose and PVA systems. Upon replacement of the solvent with [Emim]BF_4_, numerous intra‐ and intermolecular H‐bonds within PVA and cellulose are reestablished. Consequently, the crosslinking density is substantially increased, and the internal H‐bonding network becomes markedly denser (right side of Figure 1a). This robust network significantly enhances the macroscopic mechanical properties and structural stability of the gel. As shown in Figure 1b, the ionogel exhibits both flexibility and high light transmittance, can be bent or twisted to arbitrary angles, and simultaneously possesses outstanding mechanical stability, high‐temperature resistance, and electrical properties (Supporting Information S1: Figures S1 and S2).

These characteristics enable the Cel/PVA‐EB gel to simultaneously satisfy the requirements for high mechanical stability and excellent electrochemical performance, making it an ideal soft material for integrated flexible electronic devices (Figure 1c,d). In flexible supercapacitors, the Cel/PVA‐EB gel can function as an electrolyte that provides both ionic conduction and mechanical support. Its wide electrochemical window and high ionic conductivity contribute to enhanced energy‐storage performance. In smart sensors, the Cel/PVA‐EB gel responds sensitively to various external stimuli while providing stable signal output. As shown in Figure 1e, the Cel/PVA‐EB gel exhibits a combination of excellent mechanical properties, high ionic conductivity, a wide operating‐voltage window, and outstanding high‐temperature resistance.

### 2.2 Molecular network reconstruction

**FIGURE 2:**
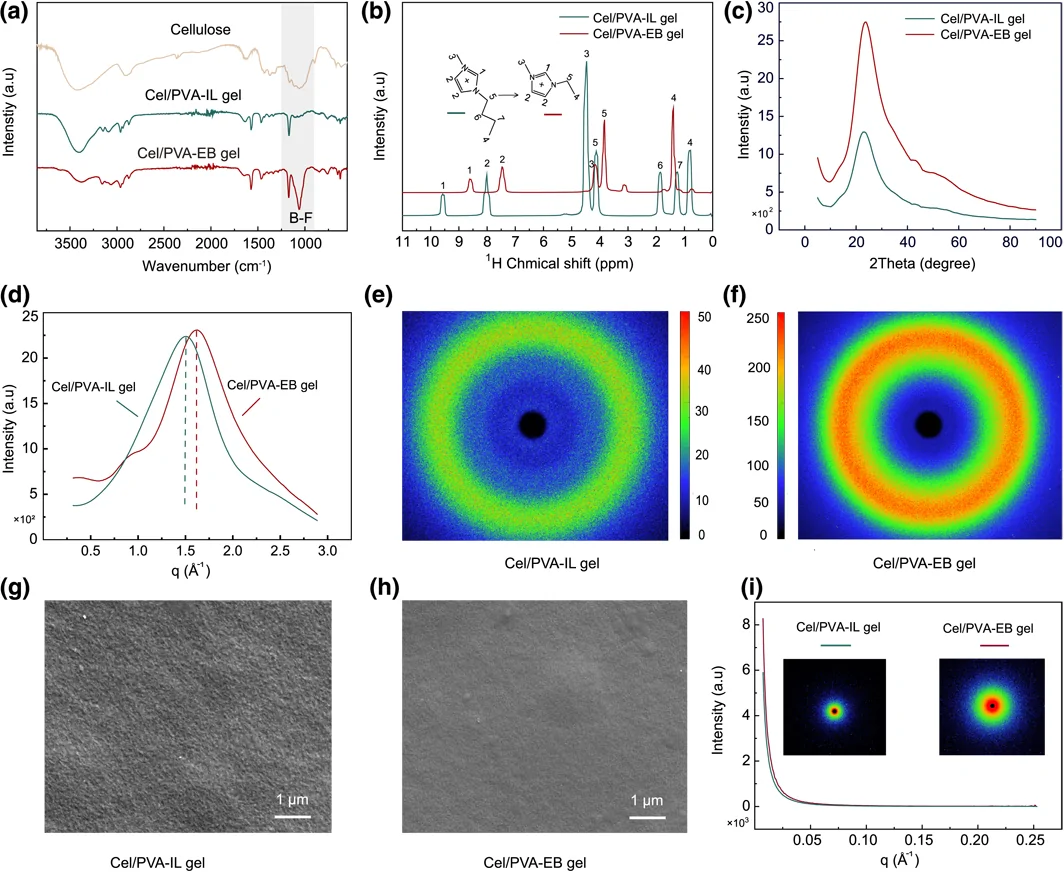
Structural characterization of cellulose‐based composite gels. (a) FT‐IR spectra of Cel‐IL, Cel/PVA‐IL gel and Cel/PVA‐EB gel. (b) ^1^H NMR, (c) XRD, and (d) wide‐angle X‐ray scattering spectra of Cel/PVA‐IL gel and Cel/PVA‐EB gel. (e) Comparison of WAXS 2D patterns of Cel/PVA‐IL gel and (f) Cel/PVA‐EB gel. (g) SEM images of Cel/PVA‐IL gel and (h) Cel/PVA‐EB gel networks. (i) Comparison of the integrated small‐angle X‐ray scattering profiles of Cel/PVA‐IL gel and Cel/PVA‐EB gel; the insets show the corresponding 2D scattering patterns.

Fourier‐transform infrared spectroscopy (FT‐IR) was employed to characterize the Cel/PVA‐IL gel and Cel/PVA‐EB gel (Figure 2a). The results revealed that the Cel/PVA‐EB gel exhibited a new characteristic absorption peak at a wavenumber of 1062 cm^−1^, which was attributed to the stretching vibration of B‐F bonds in tetrafluoroborate (BF_4_^−^). This confirms that [Emim]BF_4_ was successfully incorporated into the gel system. The NMR results (Figure 2b) further confirm the structural transformation. No characteristic signals of [Bmim]Cl were detected in the Cel/PVA‐EB gel, indicating complete removal of the original ionic liquid after solvent replacement. Ultimately, a ternary ionogel system consisting solely of cellulose, PVA, and [Emim]BF_4_ was obtained. To gain deeper insight into the structural features of the molecular network, we performed Raman spectroscopy, which reveals a peak at 2900 cm^−1^ (Supporting Information S1: Figure S3), indicating enhanced H‐bonding in the Cel/PVA‐EB gel and suggesting that the introduction of [Emim]BF_4_ promotes the crystallization behavior of PVA molecular chains. Meanwhile, X‐ray diffraction analysis also revealed that the Cel/PVA‐EB gel exhibited sharper crystal diffraction peaks than those of the Cel/PVA‐IL gel (Figure 2c). Collectively, these results demonstrate that, under the influence of [Emim]^+^ and BF_4_^−^ ions, the cellulose molecules in the Cel/PVA‐EB gel underwent molecular network reconstruction, leading to closer molecular packing and thus a denser network structure.

As shown in Figure 2d–f, the Wide‐angle X‐ray scattering curves and 2D patterns indicate that the Cel/PVA‐EB gel exhibits stronger diffraction peaks and a broader angular distribution range. All the above research results collectively verify that under the effect of [Emim]^+^ and BF_4_^−^, molecular network reconstruction occurs between PVA and cellulose molecular chains, accompanied by ordered rearrangement and dense stacking. This process drives the internal H‐bonding network structure of the gel to become denser and more stable. This reinforced network structure significantly improves the mechanical properties and thermal stability of the Cel/PVA‐EB gel.

Furthermore, to further reveal the evolution of microstructures, we employed scanning electron microscopy (SEM) to directly observe the differences in the gel microstructure before and after solvent replacement. Upon replacement with [Emim]BF_4_, the gel surface transforms from a loose, disordered configuration to a compact, uniform one (Figure 2g,h; Supporting Information S1: Figure S4). This transformation is ascribed to the close arrangement of cellulose chains and PVA segments within the Cel/PVA‐EB gel. Figure 2i presents the small‐angle X‐ray scattering patterns, which further confirm that the Cel/PVA‐EB gel possesses a denser structure. In addition, super depth of field microscopy (SDOF) observations reveal a smaller surface height difference for the Cel/PVA‐EB gel (Supporting Information S1: Figure S5), further corroborating the denser structure. This finding is consistent with the SEM observations, reflecting the coupling between the microscopic (pore size) and macroscopic (surface height difference) scales. This dense and stable cross‐linked network collectively endows the Cel/PVA‐EB gel with outstanding mechanical properties and thermal stability.

### 2.3 Network structure and performance evolution

**FIGURE 3:**
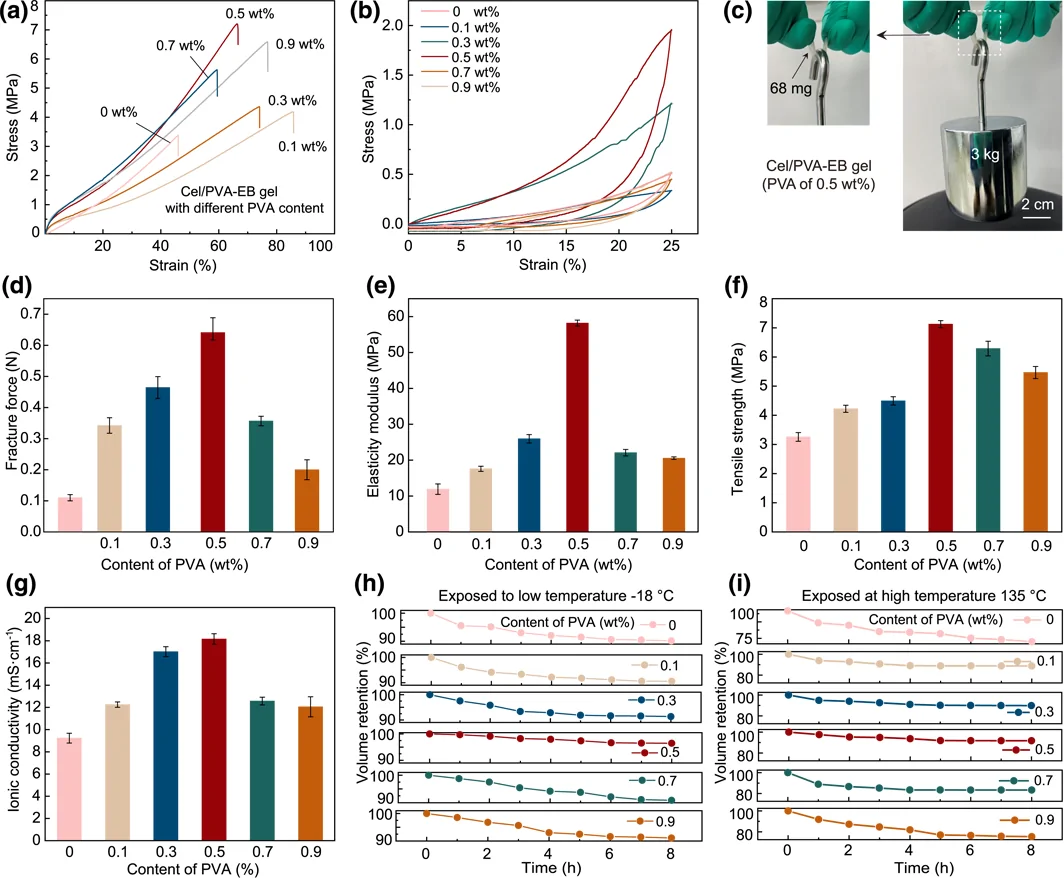
Investigating mechanical properties and ionic conductivity of Cel/PVA‐EB gel with different PVA content. (a) Mechanical tensile stress‐strain curves and (b) cyclic tensile stress‐strain curves of Cel/PVA‐EB gel with different ratios. (c) Optical image showing the Cel/PVA‐EB gel lifting a 3000 g weight. Bar charts of the (d) fracture force, (e) elastic modulus, (f) tensile strength, and (g) electrical conductivity of Cel/PVA‐EB gels with different ratios, respectively. (h) Low‐temperature and (i) high‐temperature volume retention of Cel/PVA‐EB gel with different ratios. Error bars represent SD (*n* ≥ 3).

In the reconstructed Cel/PVA‐EB double‐network structure, the interchain H‐bonds between cellulose and PVA are significantly enhanced. To systematically examine the influence of PVA content on the gel network structure and macroscopic performance, we prepared a series of Cel/PVA‐EB gels with the cellulose content fixed at 4 wt% (Supporting Information S1: Figure S6). The tensile stress‐strain curves (Figure 3a) and cyclic loading‐unloading tests (Figure 3b) reveal that the mechanical properties increase initially and then decrease with increasing PVA content, and notable differences are observed among the sample groups. At low PVA loadings, the bridging H‐bond density between PVA and cellulose is relatively low, which results in weak double‐network crosslinking and suboptimal mechanical performance. At a PVA content of 0.5 wt%, sufficient H‐bond crosslinking sites are formed within the system, and the H‐bonding network reaches a moderate compactness. As demonstrated in Figure 3c, the Cel/PVA‐EB gel of only 68 mg can support an object weighing 44,117 times its own weight (3000 g).

For samples with identical dimensions, the ionogel containing 0.5 wt% PVA shows the highest performance in fracture force, mechanical strength, elastic modulus, and ionic conductivity (Figure 3d–g). However, further increasing the PVA content to 0.7 and 0.9 wt% causes extensive agglomeration of excess PVA chains, forming an overly compact H‐bond crosslinking network. This severely restricts the mobility of the molecular chains. Under external loading, the chain segments fail to dissipate energy through the reversible rupture of H‐bonds, leading to excessively high rigidity and compromised toughness of the gel, and ultimately a pronounced decline in tensile performance.

To further verify the regulatory effect of polyvinyl alcohol (PVA) content on the weather resistance of the gel system, all Cel/PVA‐EB gels with gradient ratios were stored at −18°C for 8 h, and their volume retention rates were all higher than 90% (Figure 3h; Supporting Information S1: Figure S7). Meanwhile, in terms of thermal stability, after all samples were treated at a high temperature of 135°C for 8 h (Figure 3i; Supporting Information S1: Figures S8 and S9), their volume retention rates were all above 85%. Among them, the sample with a PVA mass fraction of 0.5 wt% achieved a volume retention rate exceeding 90%, exhibiting the optimal high‐temperature shrinkage‐resistance performance. Even under the extreme high‐temperature condition of continuous storage at 135°C for 7 days, the gel could still maintain its structural integrity, fully demonstrating its outstanding thermal stability (Supporting Information S1: Figure S8). In summary, these results indicate that incorporating PVA can simultaneously optimize the mechanical, thermal, and electrochemical properties of the ionogel. At the optimal loading of 0.5 wt%, the Cel/PVA‐EB gel forms a robust gel skeleton for enhanced mechanical performance, while offering abundant electrostatic adsorption sites to facilitate and accelerate ion diffusion.

### 2.4 Attractive mechanical performance

**FIGURE 4:**
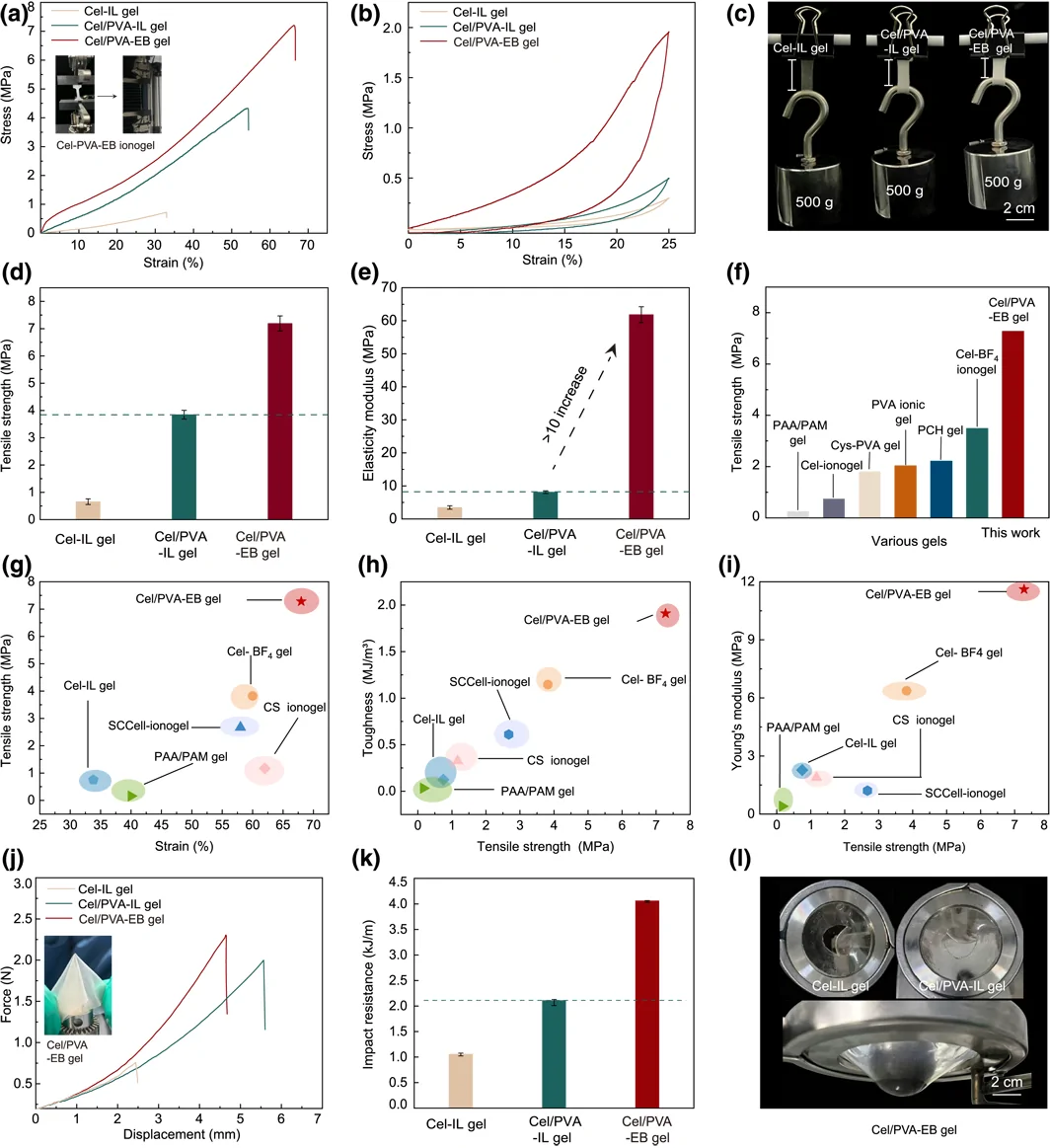
Mechanical performance comparison of Cel/PVA‐EB, Cel/PVA‐IL, and Cel‐IL ionogels. (a) Tensile stress‐strain curves (Inset: stretched shape of the Cel/PVA‐EB gel), (b) cyclic loading‐unloading curves, (c) photographs of elongation under identical tensile load, (d) tensile strength, and (e) elastic modulus. (f–i) performance comparison between Cel/PVA‐EB gel and reported ionogels: (f) tensile properties, (g) strain‐strength, (h) strength‐toughness, and (i) young's modulus‐strength. (j) Comparison of puncture resistance of Cel/PVA‐EB gel, Cel/PVA‐IL gel, and Cel‐IL gel. Inset showing the puncture morphology of the Cel/PVA‐EB gel. (k) Comparison of impact toughness of Cel/PVA‐EB, Cel/PVA‐IL, and Cel‐IL gel. (l) Photographs of the ionogel samples after impact testing. Error bars represent SD (*n* ≥ 3).

With the optimal PVA content (0.5 wt%) identified, we next systematically evaluated the comprehensive mechanical performance of the Cel/PVA‐EB gel. Upon the induction of [Emim]BF_4_, structural rearrangement occurs within the Cel/PVA‐EB gel, driving the PVA‐cellulose double network to evolve into a more compact configuration, which achieves simultaneous improvement in mechanical performance and structural stability. Compared with the single‐network Cel‐IL gel and Cel/PVA‐IL gel, the Cel/PVA‐EB gel exhibits remarkably enhanced tensile strength up to 7.20 MPa, which is 8.1 times that of the Cel‐IL gel and 1.7 times that of the Cel/PVA‐IL gel (Figure 4a).

Cyclic tensile tests reveal that under identical tensile strain, the peak tensile stress of Cel/PVA‐EB gel is far higher than that of Cel‐IL gel and Cel/PVA‐IL gel (Figure 4b). This can be attributed to the simultaneous reconstruction of bridging H‐bonds between PVA and cellulose after solvent replacement, generating abundant reversible physical crosslinking sites. Subjected to cyclic loading, these high‐density dynamic sacrificial bonds dissipate deformation energy via reversible dissociation and re‐association, thereby substantially enhancing the cyclic load‐bearing capability of the ionogel. Furthermore, profiting from the dual‐network architecture, the flexible PVA network constrains molecular‐chain slippage and cooperates with the rigid cellulose skeleton to mitigate bulk tensile deformation. Under a static tensile load of 500 g (Figure 4c), the Cel/PVA‐EB gel shows the lowest deformation magnitude, indicative of its outstanding tensile creep resistance and anti‐deformation performance.

Nanoindentation tests (Supporting Information S1: Figure S10) further corroborate the superior mechanical performance of the Cel/PVA‐EB gel. The surface hardness of the Cel/PVA‐EB gel was more than 8 times that of the Cel/PVA‐IL gel, thereby substantially enhancing the material's resistance to local indentation and plastic deformation. The densified H‐bonding network and dual‐network structure integrate rigid support with dynamic energy dissipation, thereby endowing the Cel/PVA‐EB gel with outstanding elastic recovery and deformation resistance while maintaining high tensile strength (Supporting Information S1: Figure S11). As illustrated in Figure 4d,e, the tensile strength of the Cel/PVA‐EB gel was significantly enhanced, and the elastic modulus was enhanced over 10 times compared with the state before solvent replacement. Its tensile strength surpasses that of many reported ionogel systems (Figure 4f),[^31^, ^32^, ^33^, ^34^, ^35^, ^36^] verifying that the distinctive molecular network structural design delivers superior mechanical properties. As shown in Figure 4g‐i, the Cel/PVA‐EB gel outperforms the reported ion gels in terms of tensile strength, toughness, and young's modulus.[^31^, ^32^, ^36^, ^37^, ^38^]

The comprehensive results indicate that the dual‐network structure obtained by reconstruction via [Emim]BF_4_ displacement not only possesses high strength but also enables continuous dissipation of external force, thus achieving a synergistic balance between high strength and high toughness. The puncture‐resistance performance further confirms that the Cel/PVA‐EB gel exhibits significantly superior properties compared with the Cel‐IL gel and Cel/PVA‐IL gel (Figure 4j). In the impact‐resistance test, an iron ball weighing 256 g was dropped freely from a specific height. Obvious structural damage occurred in both the Cel‐IL gel and Cel/PVA‐IL gel, whereas the Cel/PVA‐EB gel maintained structural integrity and displayed outstanding impact toughness with a value up to 4.23 kJ m^−2^, approximately twice that of the Cel/PVA‐IL gel (Figure 4k,l).

### 2.5 Thermal stability and wide‐temperature adaptability

**FIGURE 5:**
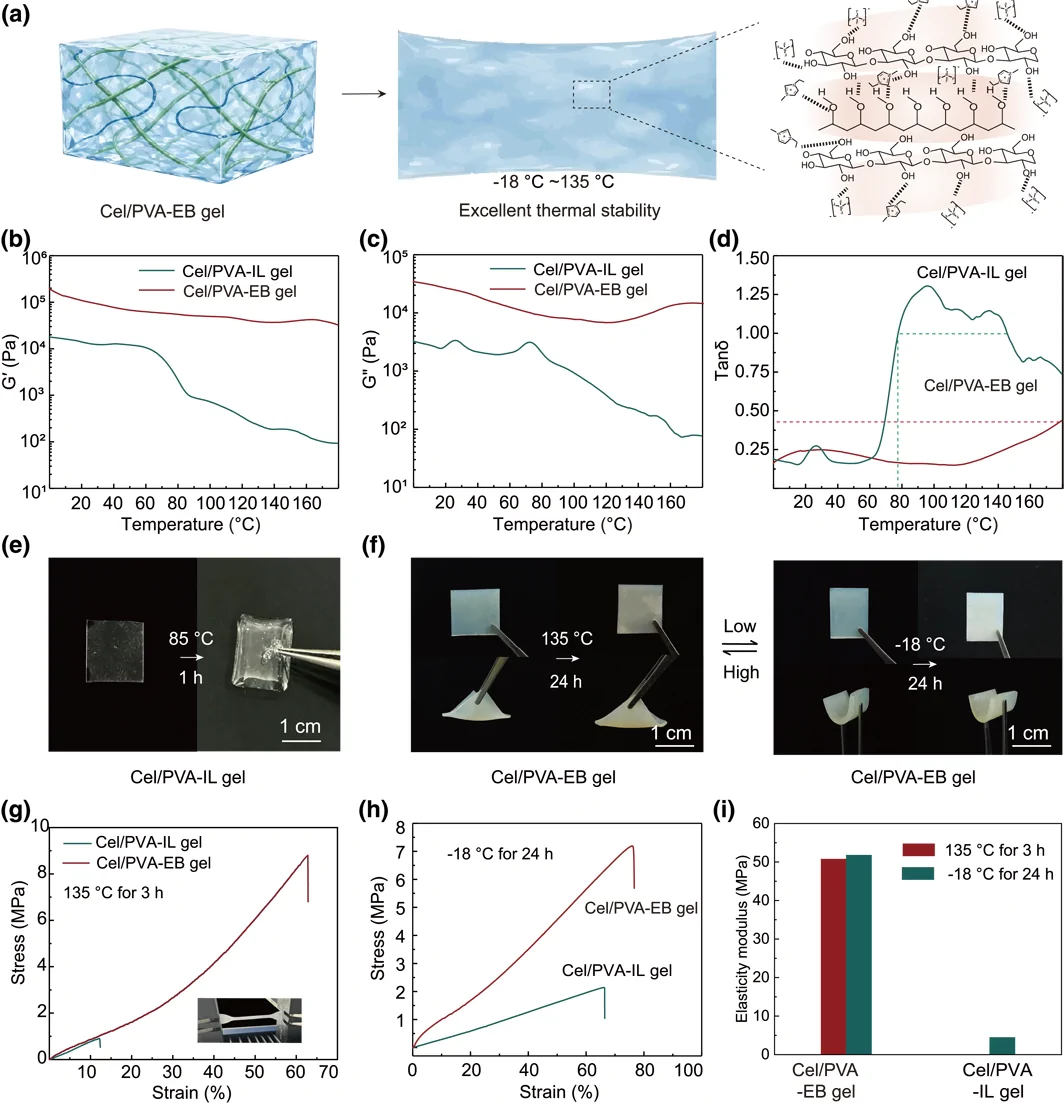
Excellent structural stability of Cel/PVA‐EB gel at both low and high temperatures. (a) Schematic diagram of environmental stability of Cel/PVA‐EB gel. (b–d) Dynamic mechanical analysis test curves of Cel/PVA‐EB gel and Cel/PVA‐IL gel: storage modulus (b), loss modulus (c), and (d) tan *δ*. (e) Digital images of Cel/PVA‐EB gel at 85°C for 1 h. (f) Digital images of Cel/PVA‐EB gel at 135°C and −18°C for 24 h. (g) Tensile performance testing of the Cel/PVA‐EB gel after 135°C for 3 h. The inset shows the sample morphology after treatment. (h) Cel/PVA‐EB gel and Cel/PVA‐IL gel were subjected to −18°C for 24 h, followed by tensile performance testing. (i) Comparison of the elastic modulus between Cel/PVA‐EB gel and Cel/PVA‐IL gel.

Benefiting from the interwoven network structure and dense H‐bonding network within the Cel/PVA‐EB gel (Figure 5a), the thermal motion of molecular chains is substantially restricted, thereby endowing the gel with excellent thermal stability. Thermogravimetric analysis (TGA, Supporting Information S1: Figure S12) revealed that the Cel/PVA‐EB gel exhibits a decomposition temperature of up to 291°C, which makes it well suited for applications in high‐temperature engineering monitoring, high‐temperature warning, and thermoelectric devices. Differential scanning calorimetry (DSC, Supporting Information S1: Figure S13) was employed to analyze the thermal behavior of the Cel/PVA‐EB gel. DSC results demonstrate that the melting endotherm of the Cel/PVA‐EB gel is significantly narrowed during the full heating‐cooling cycle, and no obvious melting transition is observed in the heating process. This further confirms that the material exhibits exceptional structural stability and dimensional integrity in the temperature range of −50°C–150°C.

Dynamic mechanical analysis results further corroborate the above conclusions (Figure 5b–d; Supporting Information S1: Figure S14). The storage modulus of the Cel/PVA‐EB gel did not show any obvious stepwise decrease over the temperature range of 0°C–165°C. More importantly, the storage modulus was consistently greater than the loss modulus throughout the entire testing temperature range, and no distinct relaxation peak was observed in the loss factor (tan*δ*) curve. This indicates that the material remains in a solid state within this temperature range, and the dense H‐bond network together with the dual‐network structure effectively suppresses the thermally activated motion of cellulose molecular chains and PVA segments during heating, with no obvious glass transition or softening occurring. This indicates that within this temperature range, the internal network structure of Cel/PVA‐EB gel remains intact, endowing it with attractive heat resistance.

After heat treatment at 85°C for 1 h, the Cel/PVA‐IL gel transformed into a viscous fluid, accompanied by complete structural collapse. In contrast, the Cel/PVA‐EB gel retained its structural integrity and excellent flexibility after exposure to 135°C or −18°C for 24 h (Figure 5e,f; Supporting Information S1: Figure S15). This fully demonstrates that the dense H‐bond cross‐linked network can suppress molecular‐chain slipping and network dissociation at high temperatures. To evaluate the mechanical properties under wide‐temperature working conditions, tensile tests were performed on Cel/PVA‐EB gels subjected to high‐temperature and low‐temperature pre‐treatments, respectively. At 135°C, the Cel/PVA‐EB gel maintained an intact structure and favorable mechanical stability (Supporting Information S1: Figures S16 and S17). After 3 h of holding, the tensile strength of the Cel/PVA‐EB gel still reached 8.80 MPa (Figure 5g). In the low‐temperature test, after storage at −18°C for 24 h, the Cel/PVA‐EB gel retained a tensile strength of 7.18 MPa (Figure 5h) and an elastic modulus of 51.74 MPa (Figure 5i). The above results verify that the Cel/PVA‐EB gel possesses outstanding mechanical stability over a wide temperature range, demonstrating its considerable application potential in high‐ and low‐temperature energy‐storage devices.

### 2.6 Electrochemical performance and applications

**FIGURE 6:**
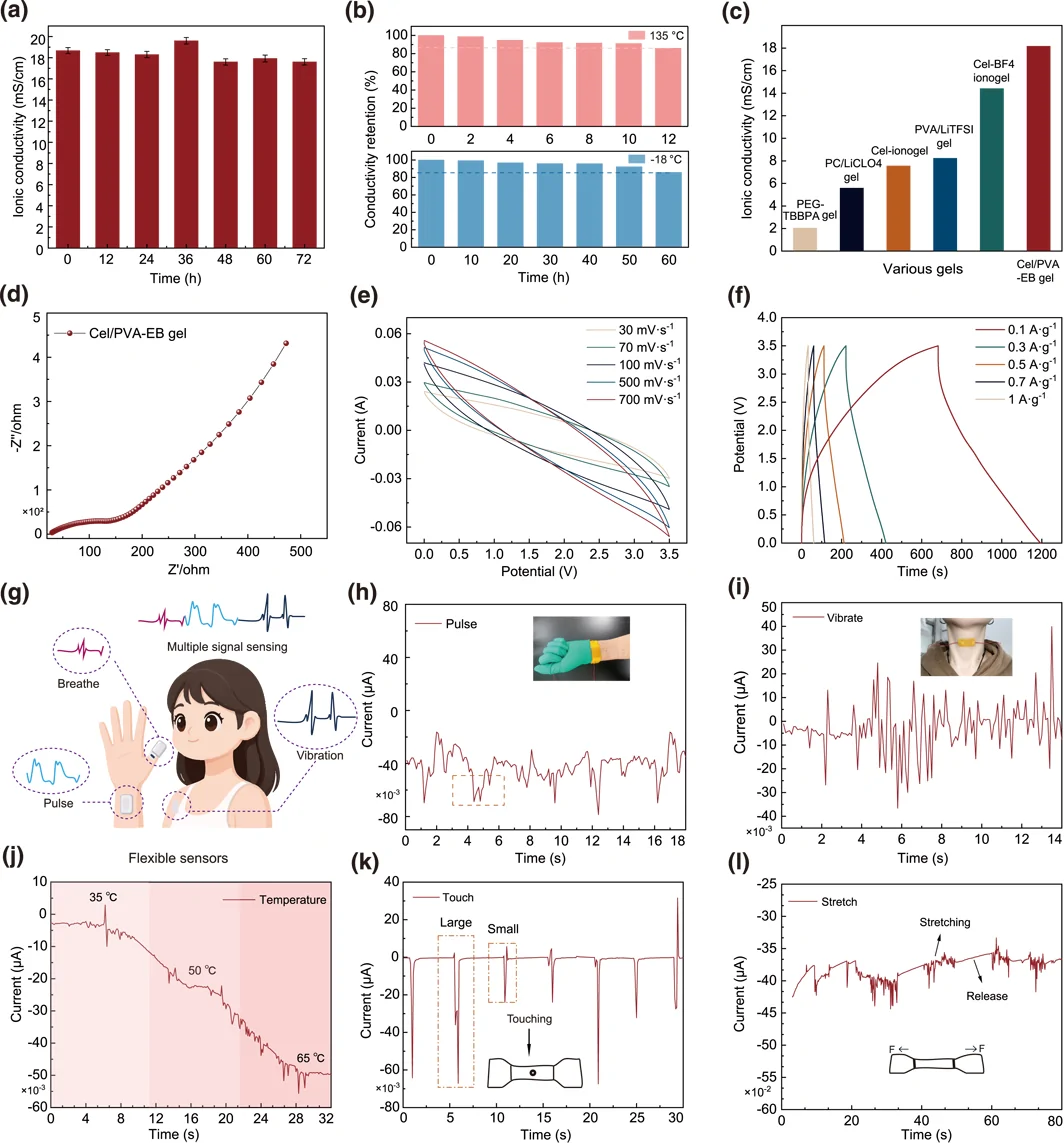
Research on the electrochemical properties and flexible sensor applications of Cel/PVA‐EB gel. (a) Investigating the ionic conductivity of Cel/PVA‐EB gel in relative humidity (RH) of 66%. (b) Retention of the ionic conductivity of the Cel/PVA‐EB gel at −18°C and 135°C. (c) Comparison of ionic conductivity between our Cel/PVA‐EB gel and previously reported ionogels. (d) Electrochemical impedance spectroscopy curves of Cel/PVA‐EB gel. (e) Cyclic voltammetry curves of the Cel/PVA‐EB gel based device at different scan rates. (f) Galvanostatic charge‐discharge curves of the Cel/PVA‐EB gel based device at different current densities. (g) Schematic diagram of a flexible sensor for signal detection. (h–l) The waveforms currently sensed by the Cel/PVA‐EB gel correspond to human pulse (h), vibration (i), temperature (j), touch (k), and stretching (l), respectively.

The Cel/PVA‐EB gel exhibits excellent environmental stability. After exposure to a relative humidity of 66% for 72 h, its ionic conductivity remains above 85% of its initial value (Figure 6a). Moreover, even after heat treatment at 135°C for 12 h or freezing at −18°C for 60 h, the ionic conductivity of the gel is still maintained above 80% (Figure 6b). Compared with the Cel/PVA‐IL gel, the electrochemical performance of the Cel/PVA‐EB gel is further enhanced. The ion transference number increases from 0.38 to 0.51, and the ionic conductivity rises from 10.7 mS/cm to 18.71 mS/cm (Supporting Information S1: Figure S18), which is superior to many reported ion gels (Figure 6c).[^39^, ^40^, ^41^, ^42^] The performance improvement is attributed to solvent‐induced displacement that drives the differential reconstruction of the H‐bonding network and electrostatic interactions inside the gel, weakens the electrostatic interactions between cellulose and ions, optimizes ion‐transport channels, and facilitates ion transport.

To further evaluate the practical application potential of the Cel/PVA‐EB gel in integrated flexible supercapacitors, we employed the Cel/PVA‐EB gel as the electrolyte and used MoS_2_ & MWCNTs along with activated carbon as the active materials to assemble a flexible supercapacitor for electrochemical testing (Supporting Information S1: Figure S19). As shown in Figure 6d, the electrochemical impedance spectroscopy curve exhibits a nearly vertical linear profile in the low‐frequency region, indicating ideal capacitive behavior and efficient ion diffusion. Figure 6e shows the cyclic voltammetry (CV) curves at different scan rates, which exhibit gradually increasing current responses with scan rate and no redox peaks, confirming stable capacitive behavior and rate capability. The CV curves are well preserved within the potential range of 0–3.50 V, indicating a stable electrochemical window. The curves in Figure 6f show good symmetry at various current densities, indicating reversible galvanostatic charge‐discharge behavior. Cycling tests demonstrate that the device retains 95.2% of its initial capacitance after 3000 cycles at a current density of 1 A g^−1^ (Supporting Information S1: Figure S20). These results indicate that the Cel/PVA‐EB gel exhibits outstanding electrochemical performance and is suitable for high‐energy‐storage applications at low current densities as well as high‐power‐output applications at high current densities.

In addition, the Cel/PVA‐EB gel also exhibits promising multi‐stimulus sensing responsiveness. Flexible sensing devices were fabricated using it as the sensing substrate, and systematic tests were carried out on its sensing response behavior. The sensor based on the Cel/PVA‐EB gel is capable of detecting multiple stimuli simultaneously and generating recognizable electrical signal feedback (Figure 6g). It enables qualitative detection of physiological signals related to human skin, including pulse (Figure 6h) and vocal‐cord vibration (Figure 6i). A distinct signal pattern can be rapidly observed for each stimulus, demonstrating the fast response and the capability of the sensor to discriminate various external stimuli. Figure 6j–l further reveal that the Cel/PVA‐EB gel sensor can also produce distinguishable signal outputs under different temperatures and mechanical stretching. Cyclic tests verify that the sensor maintains stable signals over 50 touch cycles (Supporting Information S1: Figure S21). These results confirm that the Cel/PVA‐EB gel‐based sensor delivers differentiated signal responses toward multiple stimuli, showing great potential for wearable electronics and health‐monitoring applications.

## 3 CONCLUSION

In this study, we fabricated a high‐performance ionogel by reconstructing the cellulose‐PVA supramolecular network system using [Emim]BF_4_. This reconstruction strategy achieves two molecular regulation goals, namely enhancing the H‐bonding network between cellulose molecules and reducing the electrostatic interactions between cellulose molecules and ions. The as‐prepared Cel/PVA‐EB gel exhibited excellent comprehensive properties, with a tensile strength of 7.20 MPa, an ionic conductivity of 18.71 mS⋅cm^−1^, an extreme temperature tolerance ranging from −18°C to 135°C, and a wide voltage window of 3.50 V. As a free‐standing electrolyte in flexible energy storage devices, the gel also performs remarkably. Moreover, sensors based on the Cel/PVA‐EB gel can respond to multiple stimuli and generate distinguishable electrical signal outputs, demonstrating their potential as candidate materials for further development in wearable electronics and health monitoring applications. The molecular network structure reconstruction strategy proposed in this study can effectively balance the inherent contradiction between the structural stability and multifunctionality of materials, and provides new design ideas and research approaches for the development of flexible energy‐saving bioelectronic devices.

## 4 EXPERIMENTAL SECTION

### 4.1 Materials

Cellulose, derived from wood powder with a mean degree of polymerization of ≈1500, a crystallinity of ≈64%, and a diameter of 50–200 μm, along with [Bmim]Cl ionic liquid, was obtained using reported methods.^[43]^ Multi‐walled carbon nanotubes (MWCNTs) and 1‐ethyl‐3‐methylimidazolium tetrafluoroborate ([Emim]BF_4_) were purchased from Aladdin Biochemical Technology Co., Ltd. (Shanghai, China). Activated carbon, molybdenum disulfide (MoS_2_) powder, and polyvinyl alcohol (PVA, repeat unit molecular weight of 44.05 g·mol^−1^, degree of hydrolysis of 98–99 mol%) were also obtained from the same supplier.

### 4.2 Preparation of Cel/PVA‐IL gel

A mixture of 30 g of [Bmim]Cl and 1.25 g of cellulose was stirred at 85°C for 2 h, followed by the addition of 0.16 g of PVA and further stirring for 4 h, affording a cellulose‐PVA ionogel system containing 4 wt% cellulose and (0.1, 0.3, 0.5, 0.7 and 0.9 wt%) PVA. The homogeneous system was spin‐coated onto a smooth glass plate, degassed at 85°C for 12 h, and then stored at room temperature under controlled humidity for 12 h to yield the Cel/PVA‐IL gel.

### 4.3 Preparation of Cel/PVA‐EB gel

The Cel/PVA‐IL gel was immersed in [Emim]BF_4_ at room temperature for at least 48 h to obtain the Cel/PVA‐EB gel.

### 4.4 Preparation of active materials

MoS_2_ and MWCNTs were dispersed in a certain volume of anhydrous ethanol at a mass ratio of 2:5. The mixture was ultrasonicated at 20°C for at least 10 min and then dried at 80°C for 12 h. Finally, the resulting mixture was ground for at least 30 min to obtain the MoS_2_/MWCNTs active material.

### 4.5 Fabrication of flexible cel/PVA‐EB gel‐based devices

The Cel/PVA‐IL gel was placed in an oven at 80°C for 30 min. One surface was coated with activated carbon, dried at 80°C for 10 min, and then cooled at room temperature for 2 h. Same procedure was applied to coat MoS_2_ and MWCNTs on the other side. In a glovebox, the Cel/PVA‐IL gel was immersed in [Emim]BF_4_ for 48 h. Finally, the resulting assembly was constructed into a flexible integrated energy storage device (Supporting Information S1: Figure S19).

### 4.6 Fabrication of flexible cel/PVA‐EB gel sensor

The Cel/PVA‐IL gel was placed in an oven at 80°C for 30 min. In a glovebox, the Cel/PVA‐IL gel was immersed in [Emim]BF_4_ for 48 h, and finally assembled into a flexible sensor (Supporting Information S1: Figure S22).

## AUTHOR CONTRIBUTIONS

Dawei Zhao supervised the project. Wenjuan Wang conducted most of the experiments. Wenjuan Wang wrote the paper. Changhong Lin, Minxin Wang, Jianfei Zhou and Wenjuan Wang provided critical revisions and refined the manuscript for important intellectual content. Dawei Zhao, Geyuan Jiang and Minxin Wang jointly reviewed the paper. All authors have read and approved the final submitted manuscript.

## CONFLICT OF INTEREST STATEMENT

The authors declare no conflicts of interest.

## ETHICS STATEMENT

No animal or human experiments were involved in this study.

## Supporting information

Supporting Information S1

## Data Availability

The data that support the findings of this study are available from the corresponding author upon reasonable request.
