# Supplementary material for: Cellulose supramolecular ionogel with robust mechanical strength and thermal stability for flexible electronics
Source: Smart Mol. 2026 Sep 16;4(3):e70104. doi: 10.1002/smo2.70104 (PMC13580509; doi:10.1002/smo2.70104)
Supplement: Supplementary file 1 [file SMO2-4-e70104-s001.docx]

**SUPPORTING INFORMATION**

**I. Supplementary Method**

**Characterization.** The FTIR spectra of samples were obtained by a Nicolet IS50 FTIR instrument (Thermo Fisher Scientific Inc., Waltham, MA, USA). A Bruker AVANCE Ⅲ HD 600 MHz was used to analyze the chemical structure changes of cellulose, Cel/PVA-IL gel and Cel/PVA-EB gel. The sample-to-detector distance was 2658.5 mm and exposure time 5 min. The XRD pattern of sample was measured by a D/max 2200 X-ray diffractometer (Rigaku, Tokyo, Japan) equipped with Ni-filtered Cu-Kα radiation (λ = 0.154 nm). Raman spectroscopy was performed using a high-resolution microscopic confocal Raman spectrometer (HR Evolutio) equipped with a 785 nm laser. The high-resolution microscopic confocal Raman spectrometer (HR Evolutio) was utilized to perform spatial Raman mapping. The wavelength of the excitation laser was 785 nm and the Raman mappings of the sample surface to the strength of the peak region of the characteristic peak (scan range 35 μm × 35 μm) were obtained.

The small-angle X-ray scattering (SAXS) of sample was measured using an SAXS mc^2^ instrument with Cu-Kα X-ray radiation and a wavelength of 0.154 nm (Anton Paar, Graz, Austria). The sample-to-detector distance was 1188 mm and exposure time 5 min. Wide-angle X-ray scattering (WAXS) of samples was measured using a BXeuss 2.0 SAXS (Xenocs, France). The Scanning electron microscopy (SEM) microstructure of samples was characterized by a JSM-IT800 microscope (JEOL, Tokyo, Japan) at an operating voltage of 10 kV. The rheological properties of samples were characterized using a MCR92 rheometer (Anton Paar, Graz, Austria). Oscillatory frequency sweep experiments of Cel/PVA -IL gel and Cel/PVA-EB gel were performed from 0.1 to 100 rad·s^-1^ at room temperature. The differential thermal analysis (DTA) of the samples was performed using a STA6000 thermal gravimetric analyzer (Perkin Elmer, Massachusetts, USA).

The DSC curve of sample was measured by DSC 3+ (Mettler Toledo, CH) with a temperature range of -50-150 °C. The heating rate and cooling rate both were 5 °C·min^-1^. **Rheological property test**

The rheological properties of the samples were characterized by a rotational rheometer (model: MCR92) from Anton Paar of Austria, and the dynamic viscoelasticity of the specimens at different temperatures were analyzed. The temperature slope range is 0 to 180℃, the frequency is 1Hz, and the heating rate is 10 ℃/min. The surface morphologies were observed using a DMS1000 3D ultra-depth digital microscope (Ningbo Sunny Instruments Co., Ltd., China).

**Mechanical properties test**

The tensile properties and elasticity modulus of the samples were tested by microcomputer-controlled electronic universal testing machine (Sansi Zongheng, UTM2503). The test samples size was 5 cm (length) × 1 cm (width) × 0.3-1 mm (thickness), and each group had three parallel sample strips.

For the impact resistance test of iron ball, we used a metal iron ball (256 g) as a free-falling impactor, the velocity of iron ball just touching sample was calculated by free-fall formula, *ν*_0_ = (2*hg*)^1/2^, where *h* is the free-fall distance (m) and *g* is the acceleration of gravity (9.8 m s^−2^). We adjusted *h* until the iron ball broke the samples. This was repeated three times for each material. The energy absorption of destruction of the test samples was defined by loss of kinetic energy after iron ball attacking samples. The impact resistance for specific energy absorption is calculated by (*mν*_0_^2^)/2*dρ*, where *m* is the mass of iron ball, *d* is the thickness of sample, *ρ* is the density of sample and *ν*_0_ is the velocity of iron ball touching sample. Puncture resistance test was carried out with 80.5 g steel needle.

**Mechanical testing conditions for thermally pretreated samples**

To evaluate the mechanical stability of the Cel/PVA‑EB gel after exposure to extreme temperatures, the samples were first subjected to thermal pretreatment at designated temperatures (135 °C or −18 °C) for specific durations in a convection oven or freezer. After the pretreatment, the samples were removed from the oven/freezer and allowed to equilibrate to room temperature (25 ± 2 °C) for at least 30 min prior to tensile testing. All tensile tests were performed at room temperature using a universal testing machine (Instron 5943) with a strain rate of 10 mm·min⁻¹.

**Electrochemical test**

For solid-state supercapacitor devices, the capacitances are calculated according to eq. (1):

C = $\frac{I\Delta t}{m\Delta V}$	(1)

where *I* is the discharge current (A), *Δt* is the discharge time (s), *m* is the total mass of the active substance in the device (g), and *ΔV* is the potential window.

The energy density (*E*, Wh·kg^-1^) and power density (*P*, W·kg^-1^) are calculated by eq. (2) and (3):

E = $\frac{C{(\Delta V}^{2})}{7.2}$	(2)

P = $\frac{3600E}{\Delta t}$	(3)

where *C* (F·g^-1^) is the specific capacitance of the solid-state supercapacitor device, *ΔV* (V) is the potential window, and *Δt* (s) is the discharge time.

The ionic conductivity of the ionogel is calculated as shown in the eq. (4):

σ = $\frac{L}{RA}$	(4)

Where *L* (cm) is the thickness of the samples, *R* (Ω) is the resistance of the samples, and *A* (cm^2^) is the effective contact area of the samples.

The numbers of ions transferred are calculated as the eq. (5):

$t=\frac{I_{S}(\Delta V-I_{0}R_{0})}{I_{0}(\Delta V-I_{S}R_{S})}$	(5)

The *t* (%) is transfer number of ions, *I_0_* (A) and *I_S_* (A) represent the initial current and steady-state current, respectively. *R_0_* (Ω) and *R_S_* (Ω) are the internal resistance of the initial state and the steady-state state, respectively. *ΔV* (V) is the step potential difference (10 mV).

All data are presented as mean ± SD from at least three independently prepared batches with at least three replicate measurements per batch (n ≥ 3).

**II. Supplementary Figures**

**Figure S1.** Optical images of Cel/PVA-EB gel.

**Figure S2.** Digital images of Cel/PVA-EB gel demonstrate its flexibility, transparency, and ability to be folded and twisted

**Figure S3.** Raman testing on samples Cel/PVA-IL gel and Cel-PVA-EB gel.

**Figure S4.** SEM micrographs of Cel/PVA-IL gel and Cel/PVA-EB gel at a magnification of 3000×. (a) SEM image of Cel/PVA-IL gel. (b) SEM image of Cel/PVA-EB gel.

**Figure S5**. SDOF images of the Cel/PVA-IL gel (a) and Cel/PVA-EB gel (b).

**Figure S6.** Tensile stress-strain curves of Cel/PVA-EB gel with different cellulose contents.

**Figure S7.** Optical images of different contents of PVA evolving over time at a high temperature of -18 °C.

**Figure S8.** Optical images of different contents of PVA after 7 day at a high temperature of 135 °C.

**Figure S9.** Optical images of different contents of PVA evolving over time at a low temperature of -135 °C.

**Figure S10.** Nanoindentation test results of (a) Cel‑IL gel, (b) Cel/PVA‑IL gel, an(c) d Cel/PVA‑EB gel.

**Figure S11.** Cyclic tensile fatigue test results of (a) Cel‑IL gel, (b) Cel/PVA‑IL gel, and (c) Cel/PVA‑EB gel.

**Figure S12.** TG curves of Cel/PVA-IL gel and Cel/PVA-EB gel.

**Figure S13.** DSC curves of (a) Cel/PVA-IL gel and (b) Cel/PVA-EB gel.

**Figure S14.** Comparison of storage moduli of Cel-PVA ionogel and Cel/PVA-EB gel.

**Figure S15.** Optical images of Cel/PVA-EB gel and Cel/PVA-IL gel after being placed at -18 °C and 135 °C for 24 h.

**Figure S16.** Optical photographs of the Cel/PVA-EB gel after thermal treatment at different temperatures (from left to right).

**Figure S17.** Tensile stress-strain curves of the Cel/PVA-EB gel after thermal treatment at different temperatures.

**Figure S18.** Comparison of (a) ion transference number and (b) ionic conductivity (mS/cm) for Cel/PVA-IL gel and Cel/PVA-EB gel.

**Figure S19.** Digital photograph of the flexible Cel/PVA-EB gel-based devices.

**Figure S20.** Cycling performance of the Cel/PVA-EB supercapacitor at 1 A·g⁻¹ for 3,000 cycles.

**Figure S21.** Current signal variation of the Cel/PVA-EB gel during 50 touch cycles.

**Figure S22.** Digital photograph of the flexible Cel/PVA-EB gel sensor.
